# Echovirus 30 in Bulgaria during the European Upsurge of the Virus, 2017–2018

**DOI:** 10.3390/pathogens13020143

**Published:** 2024-02-05

**Authors:** Irina Georgieva, Asya Stoyanova, Savina Stoitsova, Lubomira Nikolaeva-Glomb

**Affiliations:** 1 National Reference Laboratory for Enteroviruses, Department of Virology, National Centre of Infectious and Parasitic Diseases, 1233 Sofia, Bulgarianrl.enterovirus@ncipd.org (L.N.-G.); 2 Department of Epidemiology, National Centre of Infectious and Parasitic Diseases, 1504 Sofia, Bulgaria

**Keywords:** echovirus, meningitis, sequencing

## Abstract

In 2018, an increase in echovirus 30 (E30) detections was reported in some European countries. To assess the circulation and phylogenetic relationships of E30 in Bulgaria, E30 samples identified at the National Reference Laboratory for Enteroviruses, National Centre of Infectious and Parasitic Diseases, Bulgaria (NRL for Enteroviruses) in 2017 and 2018 were subjected to sequencing and phylogenetic analysis. The present study revealed that sample positivity did not significantly increase in Bulgaria during the European upsurge. E30 was identified in six patients, two of whom were epidemiologically linked. The maximum-likelihood phylogenetic tree showed that sequences from five patients belonged to the G1 lineage (clades G1a and G1b). The sequence from one patient belonged to the G2 lineage and was grouped closer to sequences from the last E30 outbreak in Bulgaria in 2012. No recombination events were detected. The European E30 upsurge in 2018 was caused by two clades, and one of them was G1. The fact that the majority of the Bulgarian samples belonged to G1 indicated that the virus was present in the country but did not cause a local upsurge. Phylogenetic and epidemiological data indicated sporadic E30 cases and a possible shift towards G1 lineage in 2017 and 2018.

## 1. Introduction

Enteroviruses are a wide-spread, diverse group of viruses. These members of the *Enterovirus* genus within the *Picornaviridae* family cause a variety of diseases including paralysis, encephalitis, hand-foot-and-mouth disease, non-specific febrile illness, neonatal sepsis-like disease, acute hemorrhagic conjunctivitis, myocarditis, respiratory diseases, and aseptic meningitis. Despite the undeniable clinical importance of enteroviruses, the real picture of their distribution in Europe and the burden of disease they cause is incomplete due to the insufficient and unstandardized surveillance systems used by different countries. In recent years, targeted research on specific enteroviruses has shed more light on their epidemiology. One of the most common potentially severe enterovirus diseases with central nervous system (CNS) involvement is aseptic meningitis. The latter is most frequently caused by echovirus 30 (E30) [1,2,3]. Studies from different countries have demonstrated that echovirus 30 can cause relatively large outbreaks [4,5,6]. In 2018, five European countries identified a possible increase in E30 infections, as seen through a rise in E30 positivity among enteroviral samples when compared to previous years. In most cases, reported signs and symptoms suggested meningitis [7]. In Bulgaria, there is no established system for monitoring enterovirus infections. The last major outbreak of infections caused by an enterovirus was detected in 2012 through an increase in viral meningitis cases. Studies confirmed that the cause of this outbreak was E30. Taking all of these points together, the detected upsurge of the E30 in Europe caused concern and showcased the importance of conducting more focused studies to understand E30 epidemiology in Bulgaria.

The National Reference Laboratory for Enteroviruses at the National Centre of Infectious and Parasitic Diseases (NRL for Enteroviruses) performs laboratory analysis, allowing for the identification of enterovirus infections in received samples through virus isolation in selective cell cultures followed by serotype differentiation with a microneutralization assay. This is the only laboratory in the country that performs these studies on enteroviruses. Since 2017, the molecular detection of enteroviruses has been added to routine laboratory tests, and this combined approach was used for all cases with CNS involvement and ambiguous isolation results. In 2017 and 2018, the NRL for Enteroviruses, in collaboration with the European Non-Polio Enterovirus Network (ENPEN) and Prof. dr. H. G. M. Niesters from University Medical Center, Groningen, was able to amplify these methods with further analysis of samples, including sequencing and phylogenetic analysis. In this study, variations on E30 positivity over time among enterovirus-positive patients, identified at NRL via virus isolation, were summarized. Additionally, the phylogenetic relationships of the echovirus 30 strains detected in Bulgaria with strains circulating in other parts of the world were described. These include the strains identified in the last E30 outbreak in Bulgaria in 2012 [8]. The study sheds more light on E30 epidemiology in Bulgaria over recent years.

## 2. Materials and Methods

### 2.1. Sample Collection and Flow

NRL for Enteroviruses is responsible for testing samples received via the sample flow mechanism under the case-based acute flaccid paralysis (AFP) surveillance system in the country. According to national legislation in Bulgaria, all AFP cases in children less than 15 years of age are notifiable, and samples are forwarded to NRL for Enteroviruses, where they are tested both for polio and non-polio enteroviruses. The case definition of AFP, used by Bulgarian health authorities, includes facial nerve palsy. Additionally, in Bulgaria, all cases of viral meningitis and meningoencephalitis are notifiable and samples from possible and probable viral meningitis and meningoencephalitis cases can be sent to NRL for Enteroviruses upon decision by the treating physicians. NRL also receives additional samples sent in by hospitals based on suspicion of enteroviral infection. All samples received at NRL for Enteroviruses were tested using viral isolation and microneutralization.

Additionally, samples from possible and probable viral meningitis and meningoencephalitis cases and other probable enteroviral infections with CNS involvement, as well as AFP cases with ambiguous isolation results, were tested using the PCR-based molecular detection method.

Samples found to be positive for E30 by any of the methods at NRL for Enteroviruses were forwarded to The Netherlands for sequencing (4 samples were sequenced at the National Institute of Public Health and the Environment and 2 samples at the University Medical Center, Groningen). The laboratory methods are described in more detail below.

### 2.2. Data Collection

Samples arrive at NRL for Enteroviruses with limited data, including age and sex.

### 2.3. Case Definition

For the analysis of positivity over time, a case was defined as any person who has tested E30-positive on virus isolation and microneutralization. For the phylogenetic analysis of 2017 and 2018 samples, a case was defined as any person who has tested positive on either virus isolation and microneutralization or PCR, regardless of symptoms.

### 2.4. Descriptive Analysis over Time

The total number of patients with enteroviral infection and E30 infection identified through virus isolation and microneutralization between 2017 and 2018 was described. Annual E30 percent positivity (cases (patients/contacts) with E30 as percent of all cases with non-polio enterovirus) was calculated and displayed over time.

### 2.5. Virus Isolation and Neutralization

Virus isolation was performed on all samples received, on the RD cell line in accordance with WHO protocol [9]. Briefly, culture tubes with confluent monolayers of RD cells were changed to maintenance medium and inoculated with 0.2 mL specimen extract and incubated for 5–7 days at 37 °C and 5% CO_2_ atmosphere. Cultures were examined daily using an inverted microscope for the appearance of a cytopathic effect. Microneutralization assay with equine, mixed hyper-immune antisera (RIVM enterovirus serotyping pools, National Institute of Public Health and the Environment, The Netherlands) was performed for serotype differentiation of samples with specific cytopathic effect (CPE) in the RD cell line. This assay was performed with sets of RIVM enterovirus-typing antisera that contain anti-enterovirus pools allowing the identification of coxsackievirus B1–6 and coxsackievirus A9 and 20 echoviruses (including echovirus 30). Briefly, 50 µL previously diluted (1:20) anti-enterovirus pools were incubated with 50 µL appropriate dilution of the RD-positive samples for 2 h at 37 °C and 5% CO_2_. Subsequently, 100 µL of RD cell suspension of approximately 1.5 × 10^−5^ cells per mL was added. Cell monolayers were incubated at 37 °C and 5% CO_2_ atmosphere and cytopathic effects were scored daily by inverse light microscopy for 5–7 days. The virus was identified by the pattern of inhibition of CPE by antiserum pools [9].

### 2.6. Extraction and Nucleic Acid Detection

For the molecular-based diagnostics, viral RNA was extracted from 400 μL stool suspensions, CSF, or nasopharyngeal swabs by automated extraction with ExiPrep Dx Viral DNA/RNA kit (Bioneer, Daejeon, Republic of Korea) according to the manufacturer instructions.

For reverse transcription, 10 µL of extracted RNA was denaturated at 95 °C for 5 min. cDNA was generated by adding a 7.5 µL mixture, containing 0.5 µL dNTP mix (25 mM each) (Thermo Scientific™), 0.5 µL random hexamers (10 µM), 0.5 µL 100 mM Dithiothreitol (DTT), 3.5 µL 5× First strand reaction buffer and 0.5 µL SuperScriptIII reverse transcriptase (Invitrogen, Waltham, MA, USA) at final volume of 17.5 µL. The temperature conditions for reverse transcription were as follows: 50 °C for 60 min, followed by 95 °C for 5 min.

PanEV primers were used for enterovirus detection as described previously [9], using OneTaq Hot Start Quick-Load 2× Master Mix (New England Biolabs, Ipswich, MA, USA). Briefly, 2.5 µL cDNA was added to a mixture consisting of 12.5 µL 2× Master Mix and 0.5 µL of each forward and reverse primer (10 µM) at a final volume of 25 µL and the following temperature protocol was used: 94 °C for 30 s, 30 cycles of 94 °C for 30 s; 55 °C for 45 s; 68 °C for 1 min, and final step of 68 °C for 5 min. Enterovirus nucleic acid was detected after visualization of the PCR products by agarose gel electrophoresis.

### 2.7. Sequencing and Phylogenetic Analysis

A total of six E30-positive cell culture isolates were subjected to next-generation sequencing of the complete genome or a part of the VP1 gene at NIPHE, Netherlands. In more detail, these were cell-culture isolates from fecal samples coming from two separate cases identified in 2017, and from four samples coming from two cases identified in 2018 (a fecal sample and CSF sample from each case).

Additionally, two PCR-positive (cell culture-negative) fecal samples from two patients with suspected E30, identified in 2017, were forwarded for direct sequencing of a part of the VP1 gene at the Laboratory of Clinical Virology at the University Medical Center Groningen, Netherlands. Both came out to be E30 after sequencing.

As a result, we obtained a total of eight sequences from six patients (four sequences from four patients in 2017 and four sequences from two patients in 2018).

The 5′-end nucleotide sequence of E30 VP1 (306 bp in length, nucleotide position 2586–2891 according to the reference human E30 strain Bastianni, AF311938) was selected for this study. Multiple sequence alignment was performed with Mega X [10] via the MUSCLE algorithm. The best substitution model was selected as the one with the lowest Bayesian information criterion score via implementation in Mega X software algorithms. The selected model was the Kimura 2-parameter. A discrete gamma distribution was used to model evolutionary rate differences among sites (5 categories (+G)) [11].

For comparison of 3Dpol groupings, sequences that were complete between positions 5825 and 6364 (according to E30 strain Bastianni, AF311938) were downloaded from Genbank (17 December 2020). A phylogenetic tree was inferred as conducted for VP1 above but using a general time-reversible model. A discrete gamma distribution was used to model evolutionary rate differences among sites (+G) with 5 rate categories and by assuming that a certain fraction of sites is evolutionarily invariable (+I) [12]. The statistical significance of the phylogenies constructed was estimated by bootstrap analysis with 1000 pseudoreplicate data sets.

## 3. Results

### 3.1. Estimation of Annual E30 Positivity Rate

**Figure 1:**
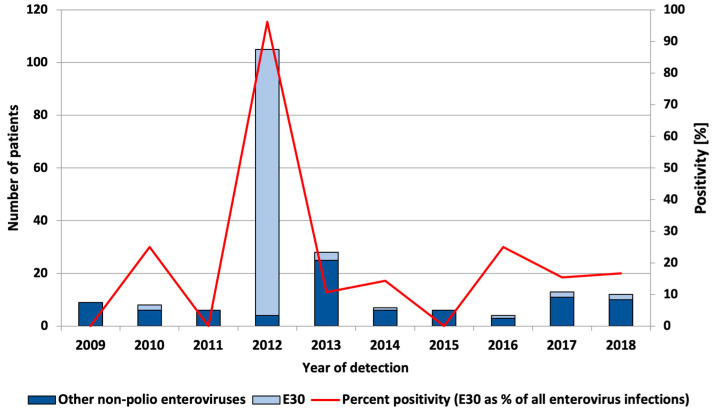
Number of echovirus 30 and other typed and untyped enterovirus infections and percent positivity of E30 infections among enterovirus infections, by year of detection.

A retrospective study of E30 positivity was conducted for eight years (from 2009 to 2018) before the European upsurge of the virus (in 2018). In 2012, an outbreak of E30 was identified and an active case finding resulted in the identification of a large number of E30 infections. The E30 positivity rate among patients/contacts whose samples were sent to NRL from this outbreak (*n* = 85) was 100%. Among enterovirus-positive patients without a clear epidemiological link to the outbreak (*n* = 20), the E30 positivity rate was 80%. The total enterovirus positivity over the period from 2009–2018 was 3.6%, and the E30 positivity rate was 2.1%. The median annual E30 percent positivity among enterovirus infections was 14.8%, with an annual positivity ranging from 0 to 96 and no clear upward or downward trend through the years was observed (Figure 1).

### 3.2. Virus Detection

Samples from a total of 799 people from different regions in Bulgaria were received at the NRL for Enteroviruses in 2017 and 2018 (408 and 391 people, respectively, in 2017 and 2018). Of those, 474 (59%) were received through AFP surveillance, 152 (19%) were possible/probable viral meningitis/meningoencephalitis cases, and 173 (22%) were sent because of suspected enteroviral infection.

Samples from all 799 people (fecal samples and/or CSF, and/or nasopharyngeal swabs) were tested via the classical virus isolation technique. Non-polio enteroviruses were detected in 25 (25/799; 3%) of the tested individuals. Echovirus 30 was identified in four (4/25, 16%).

In addition, all specimens from possible/probable viral meningitis/meningoencephalitis cases and other possible enteroviral infections, as well as specimens with a questionable result from isolation, were tested by RT-PCR (a total of 348 samples from 297 individuals). Among them, enterovirus RNA was detected via RT-PCR in 22 individuals (22/297, 7.4%).

In summary, a total of 799 unique individuals were investigated for enterovirus infection by virus isolation and 297 patients were tested both by virus isolation and RT-PCR. Among them, 47 (5.9%) were confirmed as enterovirus positive (seven by viral isolation alone; 22 by RT-PCR alone, and 18 by both methods), and six (0.75%) were confirmed as E30 positive (two by viral isolation alone, two by RT-PCR followed by sequencing, and two by both methods).

### 3.3. Patient Characteristics—2017 and 2018

Of the 47 individuals positive for enterovirus via virus isolation, RT-PCR, or both, seven (15%) were AFP patients, 22 (47%) had meningitis/meningoencephalitis/encephalitis, samples from 13 (28%) individuals were received for other reasons, and five (11%) were asymptomatic contacts or without a specified diagnosis. Of the six finally positive for E30, one was an AFP patient (a child with facial nerve palsy), four had meningitis, and one was an asymptomatic domestic contact of a known meningitis case. Thus, among the possible/probable meningitis/meningoencephalitis cases tested at the NRL for Enteroviruses in 2017 and 2018, 15% were positive for non-polio enterovirus and 2.6% for E30. Among AFP patients for the same period, 1.5% were positive for non-polio enterovirus and 0.21% for E30. Among patients with other symptoms, 7.5% were positive for non-polio enterovirus and none for E30. The clinical features of the patients tested are described in Table 1.

The median age of the cases positive for enterovirus was 14 years (range 3 months to 84) and median age of the cases positive for E30 was 19 (range 2–30).

Among E30-positive patients, three out of six were hospitalized due to meningitis in two different hospitals in Sofia without an epidemiological connection with each other. One was an AFP patient from Plovdiv without data on hospitalization. For two of the E30-positive patients, there was a clear epidemiological connection as they were living in one household. One of them was hospitalized for meningitis, and the other was asymptomatic.

### 3.4. Molecular Typing and Phylogenetic Analysis

Molecular typing of the echovirus 30-positive (typed by virus isolation) samples (*n* = 6) was performed by nucleotide sequencing of the 3′ regions of the VP1 gene. In addition to these samples, a “blind” sequencing of the same region was performed for two PanEV PCR-positive samples. A total of eight sequences from E30-positive patients (*n* = 6) were subjected to phylogenetic analysis. For two patients, the fecal sample and CSF were sequenced as individual samples as they both were RT-PCR positive.

All the obtained sequences confirmed that they belong to the E30 genotype, as they showed a >80% nucleotide similarity with the reference strains Bastianni (AF311938). The sequences were compared with those included in the GenBank database. A phylogenetic analysis in the 3′-region of VP1, at nucleotide positions 2586–2891, was performed with 69 sequences. The reconstructed tree revealed that the clinical isolates under study were grouped into two distinct lineages—G1 and G2—that showed a >5% sequence divergence from one another. These sequences were all assigned to genotype GGII, according to the classification suggested by Palacios et al. [13]. Within the G1 lineage, we identified two different clades—G1a and G1b. The mean genetic distance between the two lineages, which was calculated using the Kimura two-parameter model, is 6.58% and is greater than the mean genetic distance within these two clades (5.81%), indicating the reliability of the lineage division.

**Figure 2:**
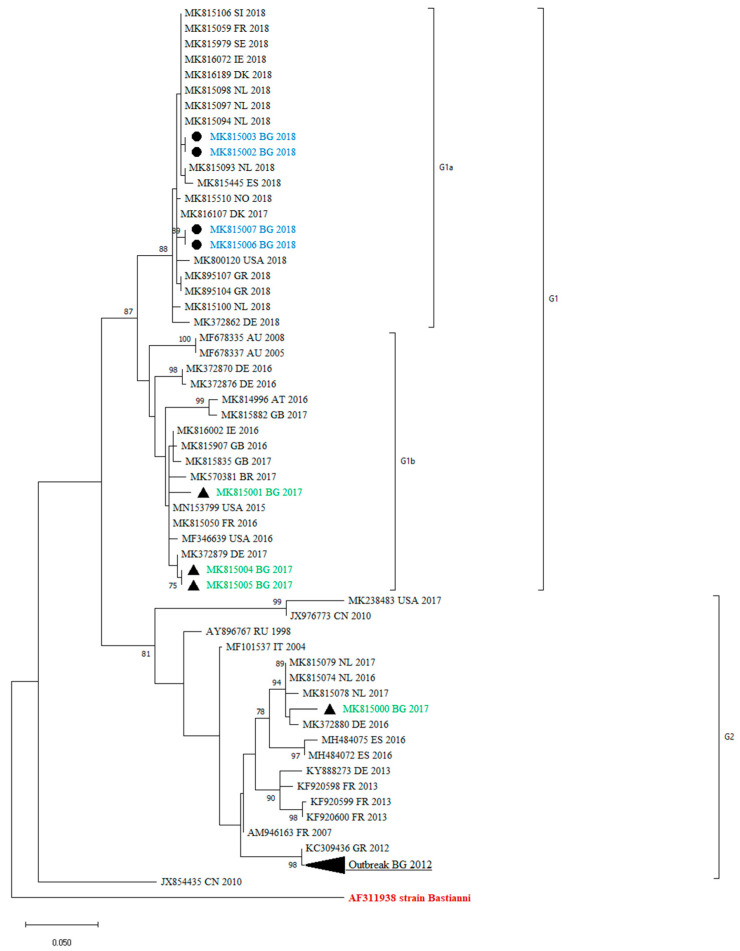
Phylogenetic analysis by maximum-likelihood method of VP1 sequences (nucleotide position 2586–2891). Clades with ≥75 bootstrap support are labeled on the branches. The tree is drawn to scale, with branch lengths measured in the number of substitutions per site. This analysis involved 69 nucleotide sequences. The cluster from the 2012 outbreak in Bulgaria is shown in black triangle. The countries of the origin of the isolates, abbreviated to two capital letters, GenBank accession numbers, and the year are indicated. MK815002 and MK815003 are two samples from one case, MK815006 and MK815007 are two samples from other case from 2018. The rest of the sequences are one sample per person.

The maximum-likelihood phylogenetic tree showed that the previously identified E30 Bulgarian isolates were grouped with one of the study sequences within G2. The remaining sequences were assigned to the G1 lineage (Figure 2).

### 3.5. Recombination Analysis

**Figure 3:**
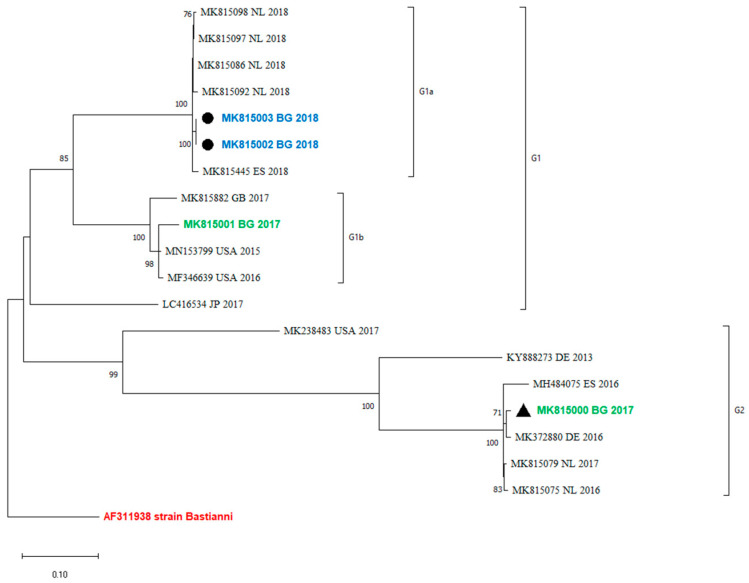
Recombination analysis of 3Dpol region (between positions 5825 and 6364) by maximum-likelihood method and general time-reversible model. The percentage of trees in which the associated taxa clustered together is shown next to the branches. This analysis involved 20 sequences. MK815002 and MK815003 are two samples from one case.

An analysis of the occurrence of recombination events between the VP1 and the 3′-distal end of the E30 genome containing the 3Dpol region was performed by a comparison of the phylogenetic grouping of these two regions. Twenty sequences that were complete between positions 5825 and 6364 were used for the analysis. The maximum-likelihood phylogenetic tree showed that viruses within VP1 clades were monophyletic in 3Dpol and did not undergo a recombination event (Figure 3).

## 4. Discussion

In Bulgaria, there is no established system for the surveillance of enterovirus infections. The only laboratory in the country responsible for enterovirus testing and typing is the NRL for Enteroviruses at NCIPD. However, some diseases, like meningitis and meningoencephalitis, which can be caused by enteroviruses, are notifiable. This makes it possible for some epidemic outbreaks to be identified. In 2012, an outbreak of aseptic meningitis was identified, and the subsequent active case finding led to the identification of over 100 patients with E30-associated disease from two Bulgarian regions [8]. Note that additionally in 2012, the E30 positivity rate among enterovirus-positive patients without a clear epidemiological link to the outbreak was particularly high, 80%, indicating a possible wider distribution of E30 during that year.

Several studies reported that E30 circulation follows a somewhat cyclical pattern [1,14,15]. To test whether the observations in our laboratory could confirm this, we performed a retrospective study of the total positivity of enteroviruses, including a period between 2009 and 2018. The total non-polio enterovirus positivity rate in the study period was 3.6%. There is no clear upward or downward trend through the years. However, these observations only include samples that were submitted to the NRL for Enteroviruses, and a large proportion of infections that actually occurred in the population probably went unnoticed.

In 2018, the Norwegian and Dutch national public health institutes observed an upsurge in the number of enterovirus-positive detections, especially echovirus 30 (E30) cases. Pursuant to these findings, the European Centre for Disease Prevention and Control (ECDC) launched a European Union (EU)-wide call to report E30 cases through the Epidemic Intelligence Information System. In most cases, reported signs and symptoms suggested meningitis [7]. Among the 15 countries contributing data, an upsurge was identified in five countries, none of which were in south or southeast Europe. In 2018, Bulgaria did not participate in the study. However, the findings from our current study show that there was no clear upsurge in E30 positivity in 2018 or the year before that (2017) (Figure 1). In 2017 and 2018, non-polio enterovirus positivity was 3% and did not differ substantially from the total non-polio enterovirus positivity (3.6%). Moreover, there has not been a clear upsurge in meningitis cases since 2012 (data not shown). Note that due to the large number of asymptomatic infections, and possible case under-ascertainment, data on positivity has to be interpreted with caution. The main limitation of our study was driven by the fact that as a National Reference Laboratory for Enteroviruses, we are responsible for polio surveillance. This means that the main activities for both polio and non-polio enterovirus testing are performed by virus isolation and microneutralization assay. Nevertheless, non-polio enterovirus positivity estimates can be informative about the general level of non-polio enterovirus infections and is a useful approach in routine ongoing surveillance to detect possible outbreaks.

In order to expand and improve our enterovirus detection capability, in recent years, we started to apply an additional molecular diagnostic for specimens from possible/probable viral meningitis/meningoencephalitis cases and other possible enteroviral infections involving CNS, as well as specimens with a questionable result from isolation. Using RT-PCR in 2017 and 2018, we confirmed an additional 22 (7.4%) EV-positive cases. The higher detection rate of viral RNA through RT-PCR is not surprising, as this is a much more efficient and sensitive detection method than cell-culture isolation. The use of a combined approach, including both diagnostic techniques, is important for a more accurate diagnosis of enterovirus infections. This practice led to the detection of E30 in one of the meningitis cases and his asymptomatic contact, which were both cell-culture negative but RT-PCR positive. Taking into account that the enterovirus carrier state among healthy individuals is common and is one of the major spreading routes for these pathogens [16,17], the investigation of contacts may be of great epidemiological importance.

In our study, we detected non-polio EVs in 22 (15%) of the 152 possible/probable meningitis/meningoencephalitis cases in 2017 and 2018. E30 was detected only in 2.6% of these cases. This low positivity suggests that E30 detections are rather sporadic, and no epidemic event is detected during the study period. Expectedly, the highest E30 positivity was observed among meningitis/meningoencephalitis patients when compared with positivity among AFP/other patients [1,2,3]. It is well known that E30 is an enterovirus that causes aseptic meningitis, often associated with outbreaks. Some authors suggest that such events occur as repeated cycles every 3–5 years [14] or at irregular intervals but with a duration of several years [15]. When compared to previous years, peaks of meningitis cases or E30 detections were not observed. The only exception is the 2012 outbreak. After that, E30 meningitis detection remains at the base level. However, it would be possible that many E30 cases could have remained undetected, due to the asymptomatic carrier state and the lack of a non-polio enterovirus surveillance system in this country.

The AFP surveillance system involves the monitoring of AFP in children less than 15 years of age, and this to some extent, distorts the median age of enterovirus-positive cases (14 years) due to the higher number of samples received from the AFP surveillance system. There was no clear difference between the median age of enterovirus patients and E30 patients, although conclusions cannot be drawn based on the small number of E30 patients included.

The epidemiological data, including hospitalizations, indicates sporadic case detection in 2017 and 2018.

Like all other enteroviruses, E30 is antigenically heterogeneous. It is divided into two genogroups designated as GGI and GGII, based on nucleotide sequence diversity in the VP1 capsid gene [13]. VP1 is the major surface-accessible protein that contributes to the antigenic neutralization sites on the surface of the enterovirus virion [18]. The VP1 sequences correlate with antigenic typing by neutralization and can be used for virus identification and evolutionary studies [19]. For that reason, we used a part of VP1 for our study. We identified two distinct lineages—G1 and G2—and two different clades within the G1 lineage—G1a and G1b. Only one of the studied viral isolates is close enough to those from the 2012 outbreak. While the 2012 isolates were very closely related to Greek isolates from the same year, the one from 2017 showed similarities with strains circulating in 2016 and 2017 in Europe (The Netherlands, Germany, and Spain). The genetic lineage G1 detected among Bulgarian isolates revealed a greater diversity. The strains identified were related to isolates from different parts of the world (Germany, the USA, Brazil, Denmark, and The Netherlands). The phylogenetic analysis confirmed the sporadic detection of E30 in Bulgaria. The close epidemic relationship is visible for the sequences from a meningitis case and his asymptomatic contact (accession numbers MK815004 and MK815005). The fact that the majority of the Bulgarian samples belonged to G1 indicated that the virus was present in the country but did not cause a local upsurge.

Many molecular epidemiology studies of E30 meningitis outbreaks reveal that outbreaks are associated with the rapid spread of different, closely related, and relatively short-lived variants [1,13,20,21]. Comparing the sequences of previously identified Bulgarian isolates with those identified in 2017 and 2018, we observed a turnover of E30 lineages, shifting from G2 to G1 (Figure 2). A similar genetic turnover was observed in Europe, where the G2 viruses that predominated in 2016 and 2017 in central Europe were subsequently replaced by the G1 and G6 viruses in 2018 [22].

Recombination is a common event among EVs and is a driving force for their evolution. Some authors observed that recombinant forms emerge, dominate, and disappear over a period of 3–5 years [14]. According to molecular studies of enterovirus evolution, recombination occurs in the region encoding non-structural genes, including the 3Dpol gene, and can be recognized by identifying the phylogenetic incongruence between the VP1 and 3Dpol gene regions [23,24,25]. This underlines the need to explore both genome regions, VP1 and 3Dpol, for the proper molecular investigation of E30 epidemics and variants. The recombination analysis in the current study showed that the 3Dpol grouping corresponds to VP1 (Figure 3). No recombination events have been observed in the study period. Unfortunately, the sequences from the last outbreak in Bulgaria are only partially sequenced and they include only a part of VP1, from nucleotide positions 2586–2891. Therefore, the occurrence of recombinations in those outbreaks and their 3Dpol grouping with more newly isolated variants cannot be tracked.

In conclusion, the present study reports data from enterovirus surveillance in Bulgaria, however incomplete it may be. The phylogenetic analysis reveals that the Bulgarian isolates belong to two genetic lineages. A turnover of E30 lineages from G2 to G1 could be hypothesized, although clearly demonstrating it would require a larger sample size.

Different variants of E30 co-circulated in the same area and caused not only meningitis, but facial nerve paralysis as well, or were asymptomatic. No recombination events have been observed in the study sequences.

It is necessary to establish an enterovirus molecular surveillance system in Bulgaria. Monitoring enterovirus infections and laboratory confirmation of the serotypes associated with different presentations may be useful in identifying the cause of a disease outbreak and can confirm that these outbreaks are not associated with preventable or currently treatable conditions, and are not associated with any novel infectious agent.

## Figures and Tables

**Table 1:** Clinical characteristics of patients tested.

Year	AFP	Meningitis/Meningoencephalitis/Encephalitis	Other
2017	250 (2 pos)	83 (15 pos)	75 (7 pos)
2018	224 (5 pos)	69 (7 pos)	98 (6 pos)

## Data Availability

E30 complete genome and VP1 sequences, reported in this study were submitted in GenBank sequence database under accession numbers MK815000-MK815007.
